# Shunting of recurrent post-traumatic syringomyelia into the fourth ventricle: a case report

**DOI:** 10.1186/1752-1947-4-210

**Published:** 2010-07-13

**Authors:** Chih-Lung Lin

**Affiliations:** 1 Department of Neurosurgery, Department of Surgery, Chang Gung Memorial Hospital and Chang Gung University, Tao-Yuan, Taiwan

## Abstract

**Introduction:**

Post-traumatic syringomyelia is a progressive degenerative disorder that is a well-recognized sequela of spinal cord injury. There is currently no optimal intervention capable of producing satisfactory long-term clinical results.

**Case presentation:**

In this report, we present a 55-year-old Asian man with recurrent syringomyelia after shunt treatment. The syrinx extended from the thoracic cord into the medulla. We used a silicone tube to create a channel connecting the syrinx cavity directly to the fourth ventricle. The patient made a good recovery and follow-up magnetic resonance imaging revealed a considerable diminution in the size of the syrinx.

**Conclusions:**

We present a new approach that has the potential to improve the outcome of patients with recurrent post-traumatic syringomyelia, who cannot be treated by conventional methods.

## Introduction

Traumatic spinal cord injury may lead to the development of syringomyelia and the incidence ranges from 0.3% to 3.2% [1]. The pathophysiology may be due to vascular, hemorrhagic, or direct mechanical trauma from the original injury, leading to formation of a cavity. Extension of the syrinx may then occur as a result of mechanical forces such as coughing and straining that transmit pressure cephalically or alternatively, from tethering of the cord due to scarring and fibrosis at the injury site [2,3]. Several treatment methods have been attempted, including shunting, laminectomy with aspiration, and syringostomy. However, they seldom yield significant functional recovery and recurrence is common. We present a case of spinal trauma complicated by the late development of syringomyelia with recurrence of the syrinx one year after shunt treatment. We introduce a new approach to re-establishing cerebrospinal flow in the subarachnoid space; following intervention, the subject of this case presentation demonstrated improved muscle power in his limbs at two-year follow-up.

## Case presentation

A 55-year-old Asian man presented with increasing neck pain and numbness of the upper extremities over the previous three months. These symptoms were exacerbated by coughing and over stretching. Six years before admission to our hospital, he had suffered a traffic accident and had sustained a spinal injury with complete paraplegia. Magnetic resonance imaging (MRI) studies at that time demonstrated a burst fracture of the L1 vertebral body with severe contusion of the spinal cord. The patient underwent a posterior fusion followed by six months of rehabilitation. He reported a definite improvement in pain sensation but remained wheelchair dependent.

Five years later, he presented with chest tightness. An MRI study revealed the presence of a syringomyelic cavity of the cord extending to the thoracic level. A T4-5 laminectomy and syringo-pleural shunt were performed in another hospital and our patient experienced a brief and intermittent improvement in chest discomfort.

**Figure 1:**
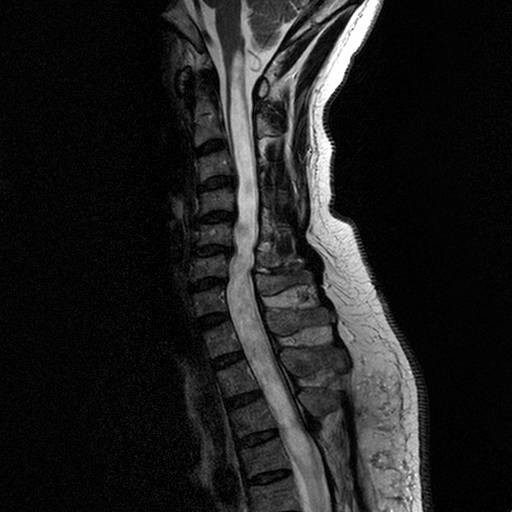
**Sagittal magnetic resonance image of the cervical spine showing the syrinx**. Although tubular hypointensity is observed on T2-weighted images, there is no connection with the fourth ventricle.

On admission to our hospital one year later, neurological examinations elicited grade III muscle strength in both upper extremities. Deep tendon reflexes were pathologically brisk in all four limbs. MRI studies of the whole spine revealed the presence of a large syrinx extending to the medulla (Figure 1).

It is likely that syrinx recurrence after the previous shunt procedure was related to shunt malfunction; moreover, it was difficult to identify any unscarred subarachnoid space. Thus, we designed a syringo-fourth ventricle shunt for this case. This procedure facilitated re-establishment of cerebrospinal fluid (CSF) flow dynamics by avoiding cord injury and damage to spinal bony structures. To the best of our knowledge, there are no previously published accounts of this procedure.

**Figure 2:**
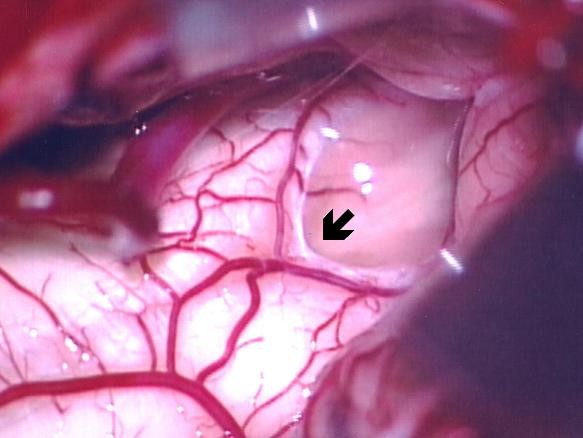
**Diagram of the dural opening and the fourth ventricle, tonsil, and posterior inferior cerebellar artery**. The obex is indicated by the black arrow.

**Figure 3:**
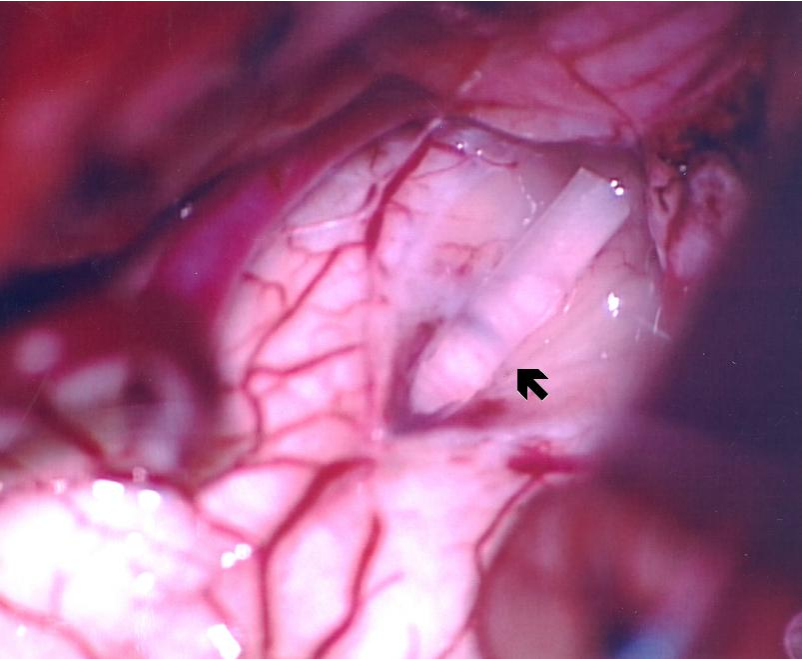
**Diagram of a silicone tube insertion (black arrow) and reconstruction of the central canal to connect the syrinx with the fourth ventricle**.

Our patient was placed in the prone position with a rigid pin-type head-holder. Suboccipital craniectomy and a partial laminectomy of C1 were performed. A Y-shaped incision was used to open the dura, and the arachnoid membrane was opened. The fourth ventricle was inspected and the obex was identified (Figure 2). Under echo guidance, we used a small ball-tip dissector to create a channel between the fourth ventricle and the syrinx. After the channel was created, high-pressure fluid ran into the fourth ventricle from the cord, which became slack. A silicone tube with a diameter of 1.5 mm and a length of 4 cm was then advanced into the syrinx cavity to a length of 2 cm. The tube was then maintained at a length of 1 cm inside the fourth ventricle (Figure 3). Tissucol fibrin sealant (Baxter, Vienna) was used to fix the tube onto the obex to avoid migration. No dural augmentation was required and the dura was repaired using water-tight sutures.

**Figure 4:**
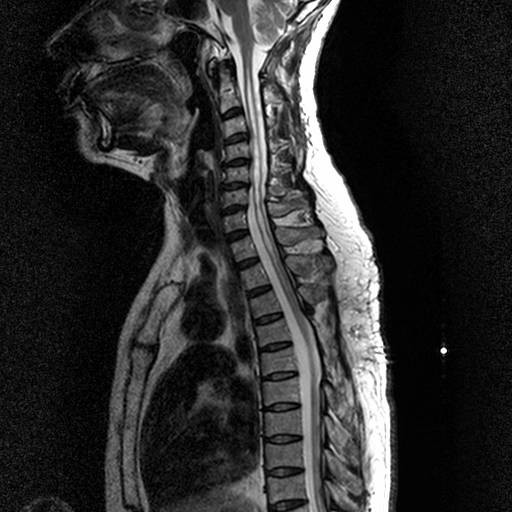
**Post-operative cervical spine MRI studies demonstrate substantial diminution in size of the syrinx on T2-weighted image**. The central canal was reconstructed using a tube.

Post-operatively, our patient complained of headache and tinnitus, which persisted for about one week and subsided spontaneously. Our patient described an improvement in numbness and pain. The muscle strength of the upper extremities improved markedly to grade IV and he gradually began to use both hands for daily activities. An MRI study performed two years after surgery showed a substantial diminution in the size of the syrinx (Figure 4).

## Discussion

Post-traumatic syringomyelia is a type of extracanalicular syrinx that originates in the spinal cord parenchyma and does not communicate with the central canal. Histological sections demonstrate hemosiderin-laden macrophages and microglia around the syrinx wall. These findings suggest that syrinx formation may result from trauma or hemorrhage [4]. For this reason, post-traumatic syringomyelia is frequently associated with significant scarring, leading to globally altered CSF flow dynamics. This makes it difficult to find unscarred subarachnoid space to insert a shunt and leads to a high recurrence rate.

Several surgical procedures have been employed to improve post-traumatic syringomyelia, including shunting to the peritoneum, the subarachnoid space or the pleural cavity; laminectomy with aspiration of the syrinx; or syringectomy. Schaller *et al. *concluded that laminectomy with arachnoid lysis and dural grafting was a promising treatment for patient with post-traumatic syringomyelia [5,6]. However, these procedures often damage spinal bony structures or result in cord injury with poor long-term outcome [7].

Our patient suffered spinal trauma with recurrent post-traumatic syringomyelia, causing delayed neurological deterioration. An MRI study of the whole spine showed that the syrinx had enlarged and extended into the medulla although a shunt had been performed one year previously. There was a practical difficultly in finding unscared subarachnoid space into which to insert the shunt. Therefore, we designed a new approach to drain the syrinx and re-establish the CSF circulation without injury to spinal bony structures or the cord. First, it is important to ensure that patients with syrinx associated with hindbrain herniation or Chiari malformation are excluded, and that a post-traumatic syrinx originates from the spinal cord parenchyma with no connection to the central canal; this avoids transmission of an incorrect CSF pulse after surgery. Second, we used a silicone tube to connect the syrinx to the fourth ventricle without injuring the already compromised cord. Finally, we observed the heartbeat-generated rhythm of fluid flow through the channel to check that the shunt was functional. A post-operative MRI study revealed a substantial reduction in the size of the syrinx, and our patient experienced a remarkable alleviation of previous symptoms.

This new approach may not be suitable for all types of syringomyelia, for example Chiari malformation-related syringomyelia, and careful patient selection is advocated. In addition, if the syrinx does not extend to the upper cervical spine, this procedure is not recommended because of the high risk of cord injury.

## Conclusions

Post-traumatic syringomyelia formation is an uncommon complication after spinal trauma and may have a delayed onset [7]. The optimal treatment for patients with syringomyelia remains controversial. In this report, we introduce a novel surgical procedure for treating post-traumatic syringomyelia by creating an intra-medullary channel to the fourth ventricle with a silicone tube; this has improved some neurological deficits in our patient in the post-operative period. However, some limitations will need to be taken into consideration; these include patient selection, operative indication and location. Although the effectiveness of our approach will need to be validated by a larger patient sample and a longer follow-up time, we believe this new approach has the potential to improve the outcome of those patients with recurrent post-traumatic syringomyelia who cannot be treated by conventional methods.

## Consent

Written informed consent was obtained from the patient for publication of this case report and any accompanying images. A copy of the written consent is available for review by the Editor-in-Chief of this journal.

## Competing interests

The author declares that they have no competing interests.
